# A candidate transporter allowing symbiotic dinoflagellates to feed their coral hosts

**DOI:** 10.1038/s43705-023-00218-8

**Published:** 2023-01-28

**Authors:** Keren Maor-Landaw, Marion Eisenhut, Giada Tortorelli, Allison van de Meene, Samantha Kurz, Gabriela Segal, Madeleine J. H. van Oppen, Andreas P. M. Weber, Geoffrey I. McFadden

**Affiliations:** 1 School of Biosciences, The University of Melbourne, Parkville, VIC Australia; 2 Institute of Plant Biochemistry, Cluster of Excellence on Plant Sciences (CEPLAS), Heinrich-Heine-University, Universitätsstraße 1, D-40225 Düsseldorf, Germany; 3 Biological Optical Microscopy Platform, The University of Melbourne, Parkville, VIC Australia; 4 Australian Institute of Marine Science, Townsville, QLD Australia; 5 Present Address: Department of Marine Biology, The Leon H. Charney School of Marine Sciences, University of Haifa, Haifa, Israel; 6 Present Address: Computational Biology, Faculty of Biology, CeBiTec, Bielefeld University, Bielefeld, Germany; 7 Present Address: Coral Resilience Lab, Hawaiʻi Institute of Marine Biology, Kāneʻohe, HI USA

**Keywords:** Symbiosis, Cellular microbiology

## Abstract

The symbiotic partnership between corals and dinoflagellate algae is crucial to coral reefs. Corals provide their algal symbionts with shelter, carbon dioxide and nitrogen. In exchange, the symbiotic algae supply their animal hosts with fixed carbon in the form of glucose. But how glucose is transferred from the algal symbiont to the animal host is unknown. We reasoned that a transporter resident in the dinoflagellate cell membrane would facilitate outward transfer of glucose to the surrounding host animal tissue. We identified a candidate transporter in the cnidarian symbiont dinoflagellate *Breviolum minutum* that belongs to the ubiquitous family of facilitative sugar uniporters known as SWEETs (sugars will eventually be exported transporters). Previous gene expression analyses had shown that *Bm*SWEET1 is upregulated when the algae are living symbiotically in a cnidarian host by comparison to the free-living state [1, 2]. We used immunofluorescence microscopy to localise *Bm*SWEET1 in the dinoflagellate cell membrane. Substrate preference assays in a yeast surrogate transport system showed that *Bm*SWEET1 transports glucose. Quantitative microscopy showed that symbiotic *B. minutum* cells have significantly more *Bm*SWEET1 protein than free-living cells of the same strain, consistent with export during symbiosis but not during the free-living, planktonic phase. Thus, *Bm*SWEET1 is in the right place, at the right time, and has the right substrate to be the transporter with which symbiotic dinoflagellate algae feed their animal hosts to power coral reefs.

## Introduction

Symbiosis is a powerful evolutionary strategy that combines skill sets from separate species to meet environmental selection challenges as a consortium. In the case of coral reefs the partnership allows abundant growth in nutrient poor waters that would otherwise support very little biomass [3]. Coral reef symbioses are remarkably successful. Although reefs occupy only 0.1% of the marine environment, they support more than 33% of marine diversity [4].

The symbiotic algae in corals use CO_2_ and light energy to manufacture sugars by photosynthesis. The sugar is fed to the coral, and this symbiotic ‘currency transfer’ [5] underpins the ability of most corals to deposit calcium carbonate and thus build a reef [3]. In turn, the coral host provides its algal symbionts with shelter, CO_2_, and precious nitrogen. Transfer of photosynthetically fixed carbon from symbiont to host was demonstrated more than 60 years ago by Muscatine and Hand [6], and symbionts provide as much as 90% of the energy used by a coral [3]. Early work, which used symbionts removed from the host, indicated that glycerol was the principal export [7]. However, recent studies using intact partnerships show that glucose is the principal export and suggest glycerol release by symbionts is either an artefact induced by isolating them from their host [8–10], or perhaps transported into the symbiosome vacuole as an osmolyte [11]. Thus, we know what form of photosynthate is transferred, and how much transfer occurs, but we know essentially nothing about how transfer occurs.

Transferred photosynthate must cross at least two membranes: the algal cell membrane; and the so-called symbiosome, a vacuole-like membrane created during the phagocytotic uptake of a symbiont by a host [12]. Given that glucose is membrane impermeable, there are probably transporters in one or both membranes to move glucose outwards. We hypothesise that at least one transporter will be in the symbiont cell membrane and will be predominantly active *in hospite*. Towards identifying the transporter(s), we previously compared gene expression in free-living and symbiotic cells of *Breviolum minutum* [1], a dinoflagellate symbiont of corals and anemones [13]. Several transporters showed heightened expression *in hospite* [1], and one—which we call *Bm*SWEET1—is further characterised here.

## Materials and methods

### Symbiotic anemone cultures

*Exaiptasia diaphana* sea anemones of genotype AIMS3 [13] were kept in a walk-in constant temperature room under the constant temperature of 26 °C, 12:12 h light:dark photoperiod cycle, and 15 μmol photons m^−2^ s^−1^ (the GBR anemones are light sensitive and prefer low light conditions [13]), along with constant air supply. Anemones were maintained in three replicate 2.4 L plastic containers with sea water (Red Sea Salt, salinity of 34 ppt), under the same conditions. Anemones were fed *ad libitum* twice a week with freshly hatched *Artemia* sp. nauplii (hatched overnight under constant air supply) and filamentous algae were removed prior to weekly full water changes.

### Culture of free-living algae

*B. minutum* cells were isolated and cultured from a single genotyped *E. diaphana* anemone [14]. Three replicate subcultures were generated in cell culture flasks (0.2 μm membrane vented cap) with 0.2 μm filtered seawater (FSW) supplemented with 1 x Diago’s IMK medium (Novachem). Cultures were kept in a growth chamber (740FHC LED, HiPoint) under a constant temperature of 26 °C, 12:12 h light:dark photoperiod cycle, and 60 μmol m^−2^ s^−1^ photons. Cultures were routinely monitored by light microscopy to assess their health (cells are intact, vital, and motile cells are present). The replicate subcultures were kept and maintained for at least six months prior to protein extractions and sampling for immunofluorescence.

### Protein sequence and prediction analysis

The amino acid sequence of *Bm*SWEET1 was deduced from the *B. minutum* transcriptome (GICE01031994) [1]. Molecular mass was calculated using the ExPASy Compute PI/MW tool (https://web.expasy.org/compute_pi/). Putative transmembrane domains were predicted using the Phobius [15] and HMMTOP [16] programs. A predicted structure of *Bm*SWEET1 was generated using ColabFold [17] and overlayed with the rice *Os*SWEET2b solved structure [18] using ChimeraX [19].

### Antiserum production

Antiserum against *Bm*SWEET1 was produced using synthetic antigenic peptides (Mimotopes) and the services of Walter and Eliza Hall (WEHI) Antibody Facility (Bundoora, VIC). Anti-*Bm*SWEET1 was raised against the peptide RKKPDENSPVSPS conjugated with keyhole limpet haemocyanin and used to immunise two different rabbits in an 82-day immunisation and bleed schedule, which included a total of four boosts. IgGs from the rabbit sera were purified using an affinity column (WEHI) and immunoreactivity (efficiency and specificity) against the antigen was assessed by ELISA (WEHI) and western blotting (see below).

### Protein extraction

Protein was extracted from *B. minutum* cultures as described [20] with the following modifications. Cells from 20 ml of culture were pelleted by centrifugation at 15,000 rpm for 1 min, then washed with FSW. A second centrifugation was followed by resuspension with FSW with 2% Triton X100 (Sigma). This step was repeated twice. The pellet was further resuspended with extraction buffer (100 mM Tris, 10 mM EDTA, 100 mM NaCl, pH 7.4) supplemented with protease inhibitor (Roche complete EDTA-free PI cocktail). The cells were homogenised using 710–1180 mm acid-washed glass beads (Sigma) and a TissueLyser II (Qiagen) for 90 s at 30 Hz. The beads were removed, and the homogenate was centrifugated at 15,000 rpm for 5 min at 4 °C. Supernatant was kept, and protein concentration determined using Qubit protein assay kit, according to manufacturer instructions (ThermoFisher).

### Western blot analysis

A protein sample of 20–30 μg was boiled (70 °C, 10 min) with Bolt LDS sample buffer (ThermoFisher) and Bolt Sample Reducing Agent (ThermoFisher) in total volume of 40–50 μl. Proteins were separated on Bolt 4–12% Bis-Tris Plus Gel (ThermoFisher) and transferred to a nitrocellulose membrane according to manufacturer’s instructions. Membrane was blocked overnight with 5% skim milk in TBS buffer (137 mM NaCl, 2.7 mM KCl, 24.8 mM Tris base, pH 7.4) containing 0.05% Tween20 (TTBS). The membrane was blotted with the primary antibody for 1 h, then washed three times with TTBS for 5 min, and then incubated with Amersham ECL anti-rabbit IgG conjugated to horse radish peroxidase (Bio-Strategy). Three additional washes of 5 min were done before exposing the membrane to SuperSignal West Pico PLUS reagents for 5 min (ThermoScientific).

To verify antiserum specificity, serum was pre-incubated with the peptide antigen (1:1 mole ratio) for 1 h with shaking at room temperature prior to western blotting as above.

### Immunofluorescence assay

Three replicates of cultured free-living and symbiotic (freshly isolated from anemones [1]) *B. minutum* cells were labelled for immunofluorescence either with anti-*Bm*SWEET1, pre-immune serum, or no primary antibody followed by washes and a secondary, fluorescent labelled antibody as below.

#### Cultured symbionts

Two ml (~1 × 10^6^ cells) from each of the three replicate flasks were sampled for quantitative immunofluorescence [16]. Cells were pelleted by spinning at 15,000 rpm, 1 min, and washed with 2 ml of FSW. Cells were fixed by incubating with 4% paraformaldehyde (Electron Microscopy Sciences) in phosphate buffered saline buffer (PBS) (137 mM NaCl, 2.7 mM KCl, 10 mM Na_2_HPO_4_, 1.8 mM KH_2_PO_4_), for 3 h at 4 °C. For permeabilization, the cells were incubated with 0.1% Triton X100 in PBS for 10 min. Cells were pelleted and washed three times with of 0.1% Tween20 (Bio-Rad) in PBS (PBST). Then blocking for unspecific binding was achieved by incubating with 1% bovine serum albumin (BSA) (Sigma) in PBST for 2 h. Further, pellet of cells was suspended with the primary antibody diluted in 0.1% BSA in PBST and incubated over-night at 4 °C. Following three washes with PBST, the cells were suspended with goat anti-rabbit IgG, Alexa Fluor 546 (ThermoScientific) 1:500 in 0.1% BSA in PBST, for 2 h, in the dark. Finally, the cells were incubated with DAPI (ThermoScientific) 1:100 in 0.1% BSA in PBST, for 10 min, in the dark.

#### Freshly isolated symbionts

Symbiont cells were isolated from 20–30 medium-sized anemones of genotype AIMS3, by homogenisation with glass homogeniser and multiple (~10) spins at 15,000 rpm, 1 min, and washes with FSW. This procedure minimised host cells in the resulting homogenate. The rest of the IFA protocol was the same as described above for the cultured symbionts.

Five µl of sample was loaded onto PTFE printed slides (SPI supplies) with ProLong™ Glass Antifade Mountant (ThermoScientific) and visualised on an inverted confocal Nikon A1R microscope. Images were acquired using DAPI and TRITC filters.

Quantification of the intensity of the signal was achieved by further analysing the images via Fiji software (ImageJ) [21], which allowed us to compare *Bm*SWEET1 signal as a proxy for protein amount. Randomly selected cells (at least 100 cells) from each of the three replicates and each treatment were identified based on their shape, size, and chlorophyll autofluorescence. Then, the mean intensity of the stain around the cell was determined based on the fluorescence from the secondary antibody. No significant difference was observed in cell wall thickness for free-living versus symbiotic algal cells (Fig. S1), so we assume that access to the labelling reagents is equivalent.

Statistical analysis for quantitative microscopy was performed using SPSS software (Version 20.0., IBM Corp). Data was tested for normality and equal variances. To distinguish statistically significant results, we used the two-way ANOVA analysis followed by post hoc (Tukey HSD) multiple-comparisons test (*p* < 0.05).

Three negative controls for immunofluorescence were performed. The first involved labelling with a pre-immune serum, which gave no signal (not shown). The second was preincubation of the serum with the peptide antigen (1:1 mole ratio) for 1 h with shaking at room temperature prior to adding the serum to slides, which resulted in no yellow *Bm*SWEET1 signal (not shown). The third negative control was to omit the primary antibody.

### Expression of *Bm*SWEET1 in yeast

The coding sequences of *At*SWEET1 (AT1G21460) and *At*SWEET11 (AT3G48740) were amplified from *Arabidopsis thaliana* cDNA using primers SF46/SF47 and SF52/SF53 (Table S1), respectively. The cDNA sequence of *Bm*SWEET1 was codon optimised for *Saccharomyces cerevisiae* and synthesised by Integrated DNA Technologies. Synthesised gene fragments were used for PCR with primers SF50/SF51. The PCR fragments were subcloned into pJet1.2 (Thermo Fisher Scientific) and verified by sequencing. After digestion with *Pac*I and *Xho*I, the fragments were ligated into the yeast expression vector pDR195 [22].

The yeast EBY4000 strain [hxt1–17D::loxP gal2D::loxP stl1D::loxP agt1D::loxP ydl247wD::loxP yjr160cD::loxP], a mutant in multiple hexose importers [23], was transformed with the different pDR195 vectors–containing either *At*SWEET1, *At*SWEET11, *Bm*SWEET1 as insert, or the pDR195 vector without insert respectively as described [24]. Transformants were selected on YNB medium + ${{{\mathrm{NH}}}}_{4^{+}}$, +His, + Trp, +2% Mal, - Ura, and verified by PCR.

To test for glucose uptake activity, the different yeast strains were precultured overnight in YNB medium (+ His, + Trp, +2% Mal) at 30 °C, 160 rpm. For EBY4000, Ura was added to the medium. Cells were harvested by centrifugation (2500 × *g*, 10 min, room temperature) and washed twice with TE buffer (10 mM Tris-HCl, pH 8.0, 1 mM EDTA). Optical density was adjusted to OD_600_ = 3. The yeast strains were spotted as a serial dilution onto YNB medium plates with or without Ura and supplemented with either 2% Mal or Glc as a carbon source. Additionally, the effect of 0.2% 2-deoxy-D-glucose was tested. Growth was documented by photography after 3 d incubation at 30 °C or 5 d incubation at 30 °C with Fru as a carbon source.

## Results and discussion

### *Bm*SWEET1 is likely a sugar transporter

**Fig. 1:**
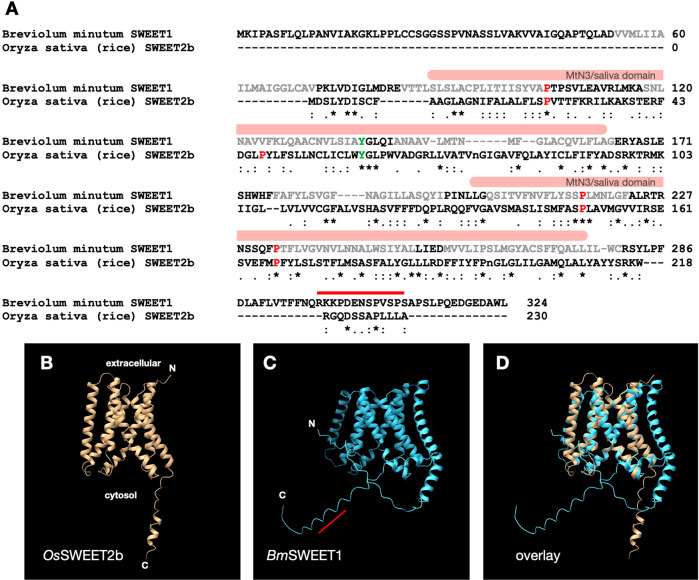
*Bm*SWEET1 has structure features of SWEET (sugars will eventually be exported transporter) sugar transporters. **A** Alignment of *Bm*SWEET1 with *Os*SWEET2b (rice SWEET2b) showing high levels of sequence similarity (* [asterisk] indicates positions with fully conserved residue,: [colon] indicates conservation between groups of strongly similar properties, and. [period] indicates conservation between groups of weakly similar properties [19]). Two *MtN3* domains in *Bm*SWEET1 are indicated by pink bars. Predicted transmembrane helices in *Bm*SWEET1 are indicated by grey text. Tyrosine 61 (green) at the extrafacial gate of *Os*SWEET2b (17) is conserved in *Bm*SWEET1, as are three of the four prolines (red) lining the *Os*SWEET2b substrate tunnel (17). The asparagine pair (lower case n) at the substrate selection site in *Os*SWEET2b (17) is not evident in *Bm*SWEET1 from this alignment. The peptide used to produce anti-*Bm*SWEET1 serum is indicated by a red line. **B** Solved structure of rice *Os*SWEET2b showing seven transmembrane helices [18]. **C** ColabFold predicted structure of *Bm*SWEET1 showing eight transmembrane domains. The approximate position of the peptide used to generate the *Bm*SWEET1 antiserum is shown as a red line. **D** Overlay of the *Os*SWEET2b (beige) and *Bm*SWEET1 (blue) structures showing extensive co-linearity plus an extra, N-terminal transmembrane domain (right hand side) in *Bm*SWEET1.

*Bm*SWEET1 (GICE01031994) has high sequence similarity to members of a transporter family known as SWEETs (sugars will eventually be exported transporters) (Fig. 1A). SWEETs were first discovered in plants [25], where their roles include exporting sugars from leaves for transport down to the non-photosynthetic roots, loading sugars into seeds to give new plants a start in life, and secretion of sugars into flower nectaries to attract pollinators [25, 26]. Since their initial discovery in plants, SWEETs have subsequently been identified in animals [27], fungi [28], protists [28, 29], and bacteria [28, 29]. Eukaryotic SWEETs apparently arose through fusion of two prokaryotic half transporters known as semi-SWEETs [28]. The structure of eukaryotic SWEETs, with two triple-helix bundles connected via an inversion linker helix (i.e., seven transmembrane domains) [18, 28], is consistent with fusion of two semi-SWEET genes [18, 28]. SWEETs are bidirectional uniporters that facilitate diffusion of sugars down a concentration gradient [25, 30]. In plants the substrates can be glucose, fructose, and sucrose [25, 30].

*Bm*SWEET1 has primary structure hallmarks of a typical eukaryotic SWEET, namely two *MtN3/saliva* domains and seven predicted transmembrane helices [18, 25]; a possible eighth transmembrane domain is predicted in an N-terminal extension of *Bm*SWEET1 not shared with plant SWEETs (Fig. 1A). Alignment [31] of *Bm*SWEET1 with the rice SWEET2b (*Oz*SWEET2b) shows a high level of sequence identity (Fig. 1A), and conserved features such as the tyrosine at the external gate [18, 25], and three of four prolines that line the transport pathway [18, 25], are evident in *Bm*SWEET1 (Fig. 1A). However, the pair of asparagine residues surrounding the glucose substrate recognition site in *Os*SWEET2b [18] is not evident in *Bm*SWEET1 (Fig. 1A). We used Alpha Fold [17] to predict a structure of *Bm*SWEET1 (Fig. 1C), and we compared the predicted dinoflagellate protein structure with the solved structure for rice, *Os*SWEET2b [18] (Fig. 1B). AlphaFold corroborated the existence of eight transmembrane domains in *Bm*SWEET1 (Fig. 1C). The predicted *Bm*SWEET1 structure is very similar the *Os*SWEET2b structure [18] with seven transmembrane helices overlapping almost exactly (Fig. 1D). The additional (eighth) predicted transmembrane in *Bm*SWEET1 protein lies to the right hand side in this view (Fig. 1C) and would result in the N-terminus being internal, which contrasts to the rice protein in which the N-terminus is extracellular [18]. What role the extra (eighth) transmembrane plays in *Bm*SWEET1, and whether *Bm*SWEET1 exists as a homotrimer like the rice glucose facilitative transporter [18], remain to be determined. Nevertheless, the similarity of the two proteins (Fig. 1D) is remarkable considering that dinoflagellates and plants last shared a common ancestor ~2b years ago [32]. Based on sequence analysis and structure modelling, we consider it extremely likely that *Bm*SWEET1 is a bidirectional sugar uniporter with indeterminate substrate preference(s).

Five additional SWEET-like genes are also evident in the *B. minutum* genomic resources [2] (Table S1). None of them were upregulated *in hospite* [1], and they are not characterised further here. Importantly, we also found SWEET-like genes in other symbiotic dinoflagellates for which genomic resources are available (Table S1).

### *Bm*SWEET1 is a cell membrane protein

**Fig. 2:**
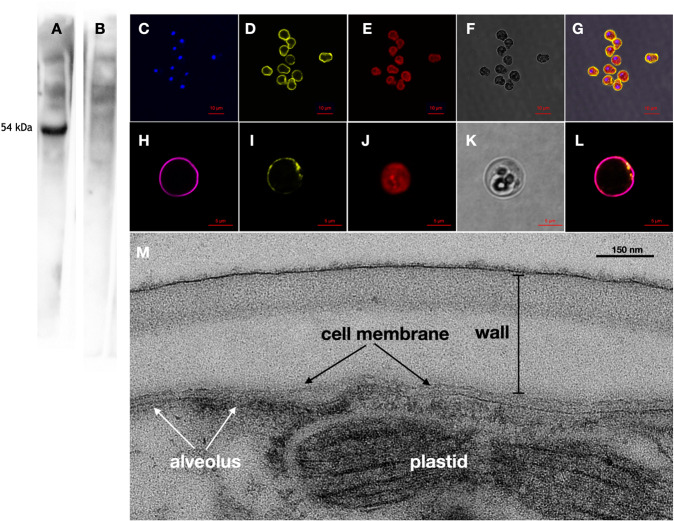
Western blot and immunofluorescence localisation of *Bm*SWEET1 to the cell membrane in *B. minutum*. **A** The peptide antiserum labels a single band of molecular mass 56 kDa in cultured cells. **B** labelling is abrogated by preincubation of the serum with the antigenic peptide. **C** Ten cultured *B. minutum* cells labelled with DAPI in blue. **D** *Bm*SWEET1 antiserum in yellow. **E** Chlorophyll autofluorescence in red. **F** Bright field illumination. **G** Merge of all, showing *Bm*SWEET1 at the periphery of the cells and not associated with the nuclei or the plastids. **H** Close up of a single cultured *B. minutum* cell stained with calcofluor for cellulose in magenta. **I** Same cell as **H** labelled for *Bm*SWEET1 in yellow, **J** Same cell as H showing chlorophyll autofluorescence in red. **K** Same cell as H in bright field illumination. **L** Merge of calcofluor and *Bm*SWEET1 showing the *Bm*SWEET1 protein mostly subtending the cellulose wall. **M** Electron micrograph of a *B. minutum* cell depicting the cell membrane situated just underneath the cell wall.

*Bm*SWEET1 is predicted to be a cell membrane protein by WoLF PSORT [33], and our next step was to localise the protein. Antisera to a peptide (RKKPDENSPVS) unique to *Bm*SWEET1 (Fig. 1A, C) decorates a single band with an apparent mass of 54 kDa in western blots of SDS-PAGE separated *B. minutum* proteins (Fig. 2A). Preincubation of the serum with the peptide abrogated binding of the serum to this band (Fig. 2B), demonstrating that the serum is specific for the *Bm*SWEET1 peptide. The apparent mass of *Bm*SWEET1 is in reasonable agreement with the predicted mass (35 kDa) considering that membrane proteins often migrate aberrantly in SDS-PAGE [34].

Immunofluorescence assays using this serum show that *Bm*SWEET1 is located at the periphery of the dinoflagellate cells, and not associated with the blue, DAPI-stained nuclei (Fig. 2C). *Bm*SWEET1 is distal to the red chlorophyll autofluorescence defining the plastids and likely not a chloroplast membrane protein (Fig. 2E–J). Co-staining with calcofluor white, which we used to stain cellulose magenta, reveals that the *Bm*SWEET1 protein is situated at or just beneath the outer cell wall (Fig. 2H–M), most likely in the dinoflagellate cell membrane. We conclude that *Bm*SWEET1 is predominantly a cell membrane (plasma membrane) protein of *B. minutum*.

### *Bm*SWEET1 transports glucose

To determine whether *Bm*SWEET1 is indeed a sugar transporter, and what its preferred substrate is, we applied the widely used yeast mutant complementation assay [18, 25, 26, 28]. The yeast hexose transporter mutant EBY4000 cannot import glucose and fails to grow on glucose agar [23]. EBY4000 can be transformed with expression plasmid pDR195, which will confer the ability to grow on glucose media if it expresses a glucose importer. Because SWEETs are bidirectional facilitators able to transport sugars from high to low concentrations, yeast expressing a functional SWEET can take up select sugars from the medium [18].

Transformants expressing *Bm*SWEET1, *Arabidopsis thaliana* SWEET1 (a glucose transporter [25]), *At*SWEET11 (an *Arabidopsis* sucrose transporter [26]), and empty pDR195 vector (containing no transporter gene) were selected on solid SD medium with 2% maltose as the carbon source at 30 °C for 4 days, then spotted as a dilution series on either maltose or glucose plates, both of which were minus uracil to select for pDR195 containing strains (pDR195 also expresses orotidine-5′-phosphate decarboxylase [URA3], which is required for uracil biosynthesis).

**Fig. 3:**
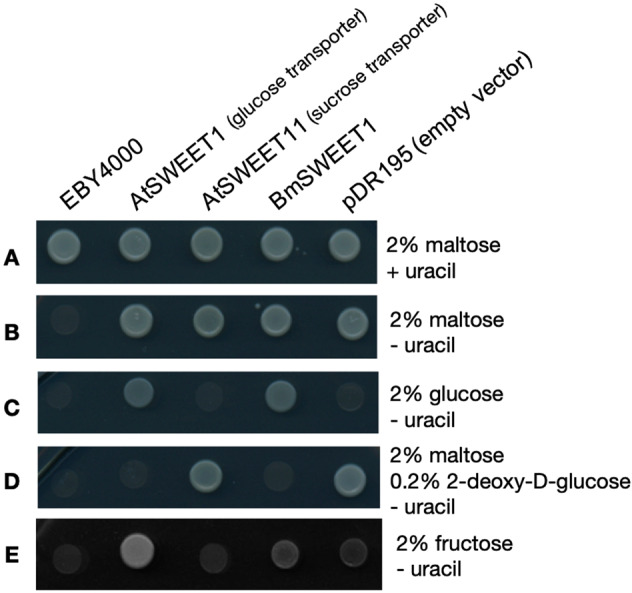
Yeast mutant complementation assay showing that *Bm*SWEET1 transports glucose and, to a lesser extent, fructose. **A** Untransformed yeast strain EBY4000 and strains transformed with the expression plasmid pDR195 harbouring the *Arabidopsis* glucose transporter *At*SWEET1, the *Arabidopsis* sucrose transporter *At*SWEET11, or the *B. minutum* putative transporter *Bm*SWEET1, or empty vector all grow on maltose (Mal) plates supplemented with uracil (Ura). **B** If uracil is omitted from maltose plates, the untransformed mutants (EBY4000) fail to grow as they are auxotrophic for uracil. **C** If maltose is replaced by glucose (Glc) only yeast expressing a glucose importer can survive, namely *At*SWEET1 transformants and *Bm*SWEET1 transformants. **D** Conversely, if maltose substrate is supplemented with a toxic glucose analogue (2-dexoy-D-glucose), any hexose import-competent strains (i.e. *At*SWEET1 transformants and *Bm*SWEET1 transformants) will die, while hexose import incompetent strains (*At*SWEET11 and pDR195 empty vector) can grow using maltose and are not poisoned by 2-dexoy-D-glucose. **E** When fructose (Fru) is supplied as the carbon source, *At*SWEET1 transformants (which can import fructose) grow, as do *Bm*SWEET1 transformants to a lesser degree. Images of the entire dilution series plates are shown in Fig S1.

All strains grew on maltose plates supplemented with uracil (Fig. 3A). Untransformed EBY4000 did not grow on maltose plates that lack uracil (Fig. 3B). As expected, the empty vector yeasts (pDR195) were able to grow on the maltose plates (Fig. 3A, B), but not glucose plates (Fig. 3C). Similarly, the *At*SWEET11 expressing yeasts grew on the maltose plates (Fig. 3A, B) but not glucose plates (Fig. 3C), which is consistent with *At*SWEET11 being a sucrose transporter [26]. Conversely, yeast expressing the known glucose transporter *At*SWEET1 [25] grew on glucose (Fig. 3C). Yeast expressing *Bm*SWEET1 also grew on glucose (Fig. 3C), confirming that *Bm*SWEET1 is a glucose transporter.

As a further test of glucose transport by *Bm*SWEET1, we employed the toxic glucose analogue 2-deoxy-D-glucose (Fig. 3D). Yeast transformants plated on maltose plus 2-deoxy-D-glucose die if they are glucose import competent. Thus, yeasts expressing *At*SWEET1 or *Bm*SWEET1 did not grow in this assay whereas those expressing *At*SWEET11, or no transporter (empty vector) grew successfully because they could not take up the toxic glucose analogue (Fig. 3D).

We also tested the ability of *Bm*SWEET1 to transport fructose (Fig. 3E). Yeasts expressing *At*SWEET1 grew on fructose as expected [35], and limited growth by yeasts expressing *Bm*SWEET1 was also evident on fructose (Fig. 3E), indicating some capacity of *Bm*SWEET1 to transport fructose. We conclude that the optimal substrate of the *Bm*SWEET1 transporter is glucose.

### *Bm*SWEET1 is more abundant in symbiotic dinoflagellates

We next sought to corroborate transcriptome evidence that *Bm*SWEET1 is more abundant when the algae are symbiotic within an animal host [1, 2] by using our antiserum to measure protein levels. Like other SWEETs, *Bm*SWEET1 is apparently an unregulated facilitator of passive diffusion, so expressing a glucose exporter in the free-living, planktonic state would appear maladaptive. Free-living cells are unlikely to export precious glucose to the ocean, and leaking glucose to the environment could also attract algivorous predators [36]. Conversely, symbiotic cells would be expected to express more *Bm*SWEET1 to fulfil their part of the symbiotic ‘currency exchange’ [5].

**Fig. 4:**
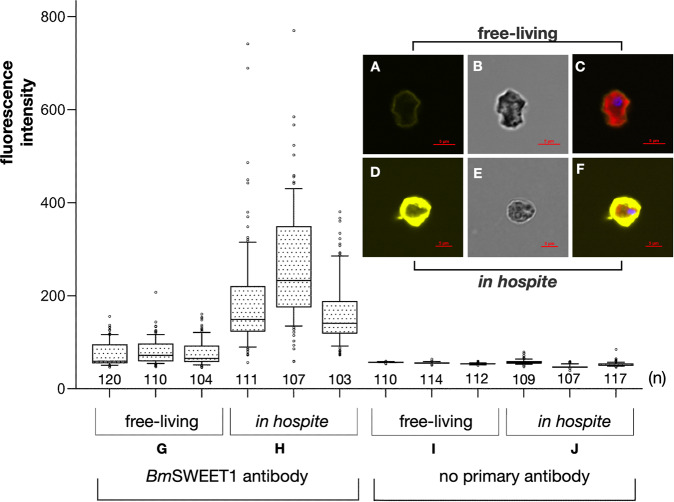
*Bm*SWEET1 is more abundant in dinoflagellates living within their host than when free-living. **A-C** *Breviolum minutum* cells cultured *in vitro*. **D**–**F** Cells of the same strain freshly isolated from host anemones. Both sets of cells were immunostained for *Bm*SWEET1 protein and imaged in parallel to compare labelling intensities. **A** *Bm*SWEET1 labelling in free-living cells showing relatively weak *Bm*SWEET1 labelling is depicted in yellow. **B** Bright field illumination. **C** Merge of all. **D** *B. minutum* cell symbiotic in a host anemone showing intense *Bm*SWEET1 labelling. **E** Bright field illumination. **F** Merge of all. **G**–**J** Immunostaining of the two populations (free-living versus *in hospite*) was performed three times, and labelling intensity was measured for >100 cells (n) and depicted as box/whisker plots. **G** Fluorescence intensity in the free-living cells. **H** Fluorescence intensity of the *in hospite* cells. **I, J** Background fluorescence intensity was quantified in equivalent numbers of cells prepared similarly but without the primary antiserum and is marginally less than was measured for free-living cells labelled with the primary antibody (compare **G**).

We attempted to measure the amount of *Bm*SWEET1 in both free-living and symbiotic algae using western blotting, but the lack of antibodies to other constitutively expressed dinoflagellate proteins to use as a loading control, and high background levels in blots of isolated symbionts (apparently caused by anemone contamination) obviated this approach. We therefore decided to quantify immunofluorescence labelling of *Bm*SWEET1 in individual cells—either free-living *B. minutum* cells, or symbiotic cells of the same strain freshly isolated from the anemone host *E. diaphana* (Fig. 4).

The antibody labelling and imaging of the two samples of algae were done in parallel with the same conditions and microscope settings (Fig. 4A–F). Labelling and quantitative comparisons were done three separate times, and fluorescent intensity of >100 cells were quantified for each treatment (Fig. 4G–J). Baseline/background labelling was established by omitting the primary *Bm*SWEET1 antiserum (Fig. 4I, J). Quantitation shows that the algae have significantly more *Bm*SWEET1 protein *in hospite* (Fig. 4H) than they do whilst free-living (Fig. 4G). To control for differential accessibility of antibodies to the free-living cells versus the freshly isolated symbiotic cells, we measured cell wall thicknesses, which showed that the median wall thickness did not differ in the two groups (Fig. S2). The symbiotic dinoflagellates exist at lower light intensity (15 μmol m^−2^ s^−1^ photons) than their free living, in vitro cultured counterparts (60 μmol m^−2^ s^−1^ photons, see Materials & Methods). To control that light intensity was not affecting the amount of *Bm*SWEET1 protein, we performed immunofluorescence on two cultures: one grown at 60 μmol m^−2^ s^−1^ photons, and another at 15 μmol m^−2^ s^−1^ photons. No significant difference in *Bm*SWEET1 immunofluorescence labelling was observed at different light intensities in vitro (Fig. S3).

Interestingly, the labelling levels in the free-living state (Fig. 4G) are barely more than the negative controls in which the primary antiserum was omitted (Fig. 4I, J), which is consistent with our hypothesis of minimal *Bm*SWEET1 expression during this life phase to minimise wasteful glucose export or danger of attracting algivores [36]. Host secreted factors are believed to induce export of fixed carbon from dinoflagellate symbionts [37–39], and various plant pathogens are able to upregulate plant SWEETs to gain access to host sugar [40], so it will be interesting to determine what causes the heightened expression of *Bm*SWEET1 *in hospite*.

We conclude that *Bm*SWEET1 is upregulated, and perhaps more stable, in symbiotic algae, which is consistent with it being an exporter of glucose able to feed its host.

## Conclusions

We focused on a putative glucose transporter, *Bm*SWEET1, in the symbiotic dinoflagellate *B. minutum* that had previously been shown to be upregulated at the gene expression level *in hospite* compared to free-living [1]. Sequence analysis and structure modelling suggested *Bm*SWEET1 is a facilitative sugar transporter that would assist sugar to diffuse down a concentration gradient. We localised *Bm*SWEET1 to the symbiont cell membrane. We showed that *Bm*SWEET1 transports glucose by complementing a hexose import deficient yeast mutant. Finally, we showed by quantitative microscopy that *B. minutum* dinoflagellates living within anemone hosts harbour substantially more *Bm*SWEET1 in their cell membranes than free-living cells of the same strain.

*Bm*SWEET1 thus has three properties making it a likely candidate for the symbiotic exporter of glucose in dinoflagellate-invertebrate symbioses: i/ appropriate location (algal cell membrane), ii/ right substrate (glucose), and iii/ markedly higher expression level *in hospite*. With dinoflagellate photosynthesis as a glucose source, and host animal respiration as a glucose sink, *Bm*SWEET1 is an ideal gateway to facilitate the diffusion of glucose from endosymbiont towards its host and thus feed corals to underpin the creation of coral reefs.

## Supplementary information

Supplemental material

## Data Availability

Materials described in the manuscript, including all relevant raw data, are freely available upon request.

## References

[1] Maor-Landaw K, van Oppen M, McFadden GI (2020). Symbiotic lifestyle triggers drastic changes in the gene expression of the algal endosymbiont *Breviolum minutum* (Symbiodiniaceae). Ecol Evol.

[2] Xiang T, Jinkerson RE, Clowez S, Tran C, Krediet CJ, Onishi M (2018). Glucose-induced trophic shift in an endosymbiont dinoflagellate with physiological and molecular consequences. Plant Physiol.

[3] Muscatine L. The role of endoysmbiotic algae in carbon and energy flux in reef corals. In: Dubinsky Z, editor. Ecosystems of the world: coral reefs. Amsterdam: Elsevier; 1990. p. 75–88.

[4] Fisher R, O’Leary RA, Low-Choy S, Mengersen K, Knowlton N, Brainard RE (2015). Species richness on coral reefs and the pursuit of convergent global estimates. Curr Biol.

[5] Wein T, Romero Picazo D, Blow F, Woehle C, Jami E, Reusch TBH (2019). Currency, exchange, and inheritance in the evolution of symbiosis. Trends Microbiol.

[6] Muscatine L, Hand C (1958). Direct evidence for the transfer of materials from symbiotic algae to the tissues of a coelenterate. Proc Natl Acad Sci USA.

[7] Muscatine L (1967). Glycerol excretion by symbiotic algae from corals and *Tridacna* and its control by the host. Science..

[8] Rees T, Fitt W, Baillie B, Yellowlees D (1993). A method for temporal measurement of hemolymph composition in the giant clam symbiosis and its application to glucose and glycerol levels during a diel cycle. Limnol Oceanogr.

[9] Ishikura M, Adachi K, Maruyama T (1999). Zooxanthellae release glucose in the tissue of a giant clam, *Tridacna crocea*. Mar Biol.

[10] Burriesci MS, Raab TK, Pringle JR (2012). Evidence that glucose is the major transferred metabolite in dinoflagellate-cnidarian symbiosis. J Exp Biol.

[11] Lin S, Cheng S, Song B, Zhong X, Lin X, Li W (2015). The *Symbiodinium kawagutii* genome illuminates dinoflagellate gene expression and coral symbiosis. Science..

[12] Davy SK, Allemand D, Weis VM (2012). Cell biology of cnidarian-dinoflagellate symbiosis. Microbiol Mol Biol Rev.

[13] Dungan A, Hartman L, Tortorelli G, Belderok R, Lamb A, Pisan L (2020). *Exaiptasia diaphana* from the Great Barrier Reef: a valuable resource for coral symbiosis research. Symbiosis..

[14] Tortorelli G, Belderok R, Davy S, McFadden G, van Oppen M (2019). Host genotypic effect on algal symbiosis establishment in the coral model, the anemone *Exaiptasia diaphana*, from the Great Barrier Reef. Front Mar Sci.

[15] Kall L, Krogh A, Sonnhammer EL (2004). A combined transmembrane topology and signal peptide prediction method. J Mol Biol.

[16] Tusnady GE, Simon I (2001). The HMMTOP transmembrane topology prediction server. Bioinformatics..

[17] Mirdita M, Schutze K, Moriwaki Y, Heo L, Ovchinnikov S, Steinegger M (2022). ColabFold: making protein folding accessible to all. Nature Methods.

[18] Tao Y, Cheung LS, Li S, Eom JS, Chen LQ, Xu Y (2015). Structure of a eukaryotic SWEET transporter in a homotrimeric complex. Nature..

[19] Pettersen EF, Goddard TD, Huang CRC, Meng EEC, Couch GS, Croll TI (2021). UCSF ChimeraX: Structure visualization for researchers, educators, and developers. Protein Sci.

[20] Weis VM, Verde EA, Reynolds WS (2002). Characterization of a short form peridinin-chlorophyll-protein (PCP) cDNA and protein from the symbiotic dinoflagellate *Symbiodnium muscatine* (Dinophycease) from the sea anemone *Anthopleura elegantissima* (Cnidaria). J Phycol.

[21] Schindelin J, Arganda-Carreras I, Frise E, Kaynig V, Longair M, Pietzsch T (2012). Fiji: an open-source platform for biological-image analysis. Nat Methods.

[22] Rentsch D, Laloi M, Rouhara I, Schmelzer E, Delrot S, Frommer WB (1995). NTR1 encodes a high affinity oligopeptide transporter in *Arabidopsis*. FEBS Lett.

[23] Wieczorke R, Krampe S, Weierstall T, Freidel K, Hollenberg CP, Boles E (1999). Concurrent knock-out of at least 20 transporter genes is required to block uptake of hexoses in *Saccharomyces cerevisiae*. FEBS Lett.

[24] Gietz RD, Schiestl RH (1991). Applications of high efficiency lithium acetate transformation of intact yeast cells using single-stranded nucleic acids as carrier. Yeast..

[25] Chen LQ, Hou BH, Lalonde S, Takanaga H, Hartung ML, Qu XQ (2010). Sugar transporters for intercellular exchange and nutrition of pathogens. Nature..

[26] Chen LQ, Qu XQ, Hou BH, Sosso D, Osorio S, Fernie AR (2012). Sucrose efflux mediated by SWEET proteins as a key step for phloem transport. Science.

[27] Artero RD, Terol-Alcayde J, Paricio N, Ring J, Bargues M, Torres A (1998). *saliva*, a new *Drosophila* gene expressed in the embryonic salivary glands with homologues in plants and vertebrates. Mech Dev.

[28] Hu YB, Sosso D, Qu XQ, Chen LQ, Ma L, Chermak D (2016). Phylogenetic evidence for a fusion of archaeal and bacterial SemiSWEETs to form eukaryotic SWEETs and identification of SWEET hexose transporters in the amphibian chytrid pathogen *Batrachochytrium dendrobatidis*. FASEB J..

[29] Yuan M, Wang S (2013). Rice MtN3/saliva/SWEET family genes and their homologs in cellular organisms. Mol Plant.

[30] Eom JS, Chen LQ, Sosso D, Julius BT, Lin IW, Qu XQ (2015). SWEETs, transporters for intracellular and intercellular sugar translocation. Curr Opin Plant Biol.

[31] Larkin MA, Blackshields G, Brown NP, Chenna R, McGettigan PA, McWilliam H (2007). Clustal W and Clustal X version 2.0. Bioinformatics..

[32] Strassert JFH, Irisarri I, Williams TA, Burki F (2021). A molecular timescale for eukaryote evolution with implications for the origin of red algal-derived plastids. Nat Commun.

[33] Horton P, Park KJ, Obayashi T, Fujita N, Harada H, Adams-Collier CJ (2007). WoLF PSORT: protein localization predictor. Nucleic Acids Res.

[34] Rath A, Glibowicka M, Nadeau VG, Chen G, Deber CM (2009). Detergent binding explains anomalous SDS-PAGE migration of membrane proteins. Proc Natl Acad Sci USA.

[35] Le Hir R, Spinner L, Klemens PA, Chakraborti D, de Marco F, Vilaine F (2015). Disruption of the sugar transporters AtSWEET11 and AtSWEET12 affects vascular development and freezing tolerance in *Arabidopsis*. Mol Plant.

[36] Chet I, Fogel S, Mitchell R (1971). Chemical detection of nicrobial prey by bacterial predators. J Bacteriol.

[37] Gates RD, Hoegh-Guldberg O, McFall-Ngai MJ, Bil KY, Muscatine L (1995). Free amino acids exhibit anthozoan “host factor” activity: they induce the release of photosynthate from symbiotic dinoflagellates in vitro. Proc Natl Acad Sci USA.

[38] Sutton DC, Hoegh-Guldberg O (1990). Host zooxanthellae interactions in four temperate marine invertebrate symbioses: assessment of effect of host extracts on symbionts. Biol Bull.

[39] Wang JT, Douglas AE (1997). Nutrients, signals, and photosynthate release by symbiotic algae (The impact of taurine on the dinoflagellate alga *Symbiodinium* from the sea anemone *Aiptasia pulchella*). Plant Physiol.

[40] Breia R, Conde A, Badim H, Fortes AM, Geros H, Granell A. Plant SWEETs: from sugar transport to plant-pathogen interaction and more unexpected physiological roles. Plant Physiol. 2021;186:836–52. 10.1093/plphys/kiab127 PMC8195505 33724398

