# Supplementary material for: A candidate transporter allowing symbiotic dinoflagellates to feed their coral hosts
Source: ISME Commun. 2023 Jan 28;3:7. doi: 10.1038/s43705-023-00218-8 (PMC9884229; doi:10.1038/s43705-023-00218-8)

Table S1. Candidate SWEET genes in symbiotic dinoflagellates

**Species**	**GenBank identifier**
Breviolum minutum	GICE01025355, GICE01018569, GICE01025163, GICE01015543, GICE01026504
Symbiodinium spp.	HBTH01040561, GAKY01013338, GBSC01010250, GBRZ01006132, GAKY01013339, GBGW01004312.1, GAKY01013337, GAKY01013336.1, HBTG01050769.1, GFDR03029040.1, CAE7348669, CAE7446774, CAE7251408.1, OLP84466.1, CAE7227003.1
Cladocopium goreaui	GBSC01011602<br>GAFO01010676<br>GBSC01033791
Durusdinium trenchii	IADN01018491<br>GBRR01018589

Table S2. Primers used in this work.

**Primer**	**Sequence (5’→ 3’)**
SF46	CACCTTAATTAAATGAACATCGCTCACACTATCT
SF47	CACCCTCGAGTTAAACTTGAAGGTCTTGCTTTC
SF50	CACCTTAATTAAATGAAAATCCCCGCAAG
SF51	CACCCTCGAGTCATAACCAGGCATCCTCTC
SF52	CACCTTAATTAAATGAGTCTCTTCAACACTGAAAAC
SF53	CACCCTCGAGTCATGTAGCTGCTGCGG

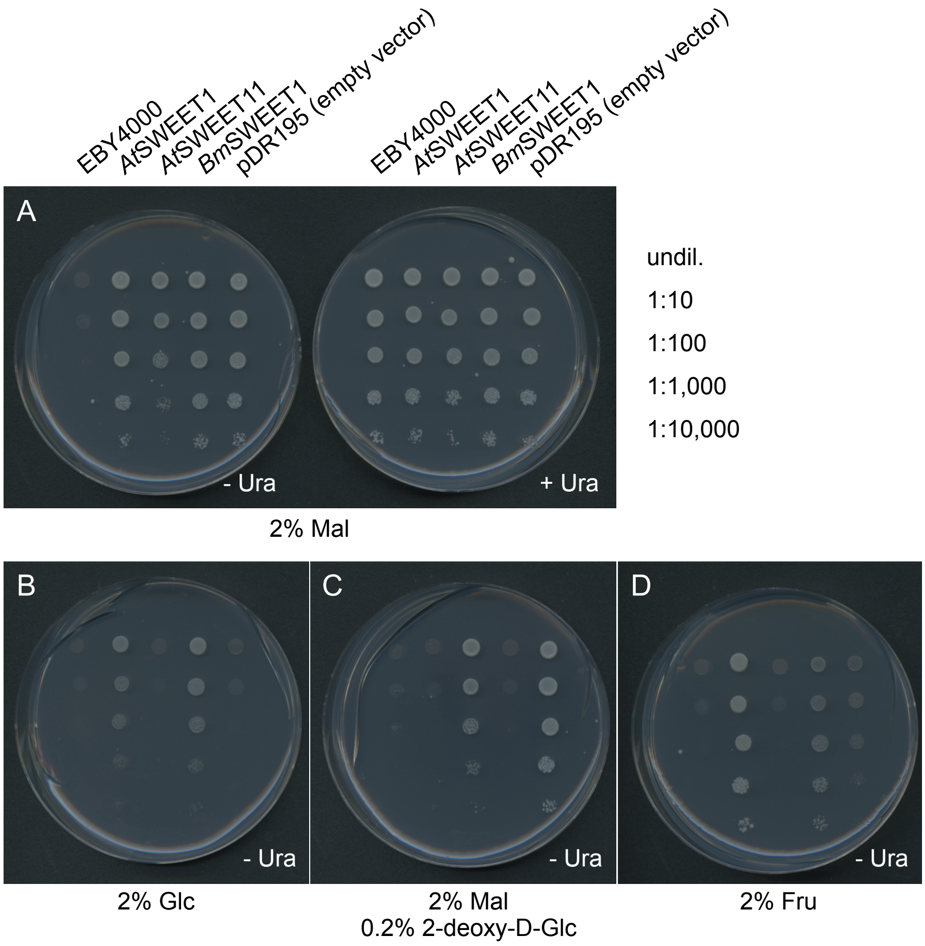

Figure S1. Images of entire plates showing dilution series of yeast mutant complementation assay depicted in Figure 3.

Cell cultures were adjusted to an OD_{600} of 3.0. For each strain, a dilution series with 1:10, 1:100, 1:1,000, and 1:10,000 was generated and 5 µL of eqach dilution was dropped onto YNB plates containing NH_4^+, His, and Trp. Uracil (Ura) was added (+) or omitted (-) from medium for selection. As carbon sources, 2% maltose (Mal, A+C), glucose (Glc, B), or fructose (Fru, D) were added. In C, medium was additionally supplemented with 0.2% of the toxic Glc analog 2-deoxy-D-Glc. Growth was documented after incubation for 3 d (5 d for Fru) at 28 °C.

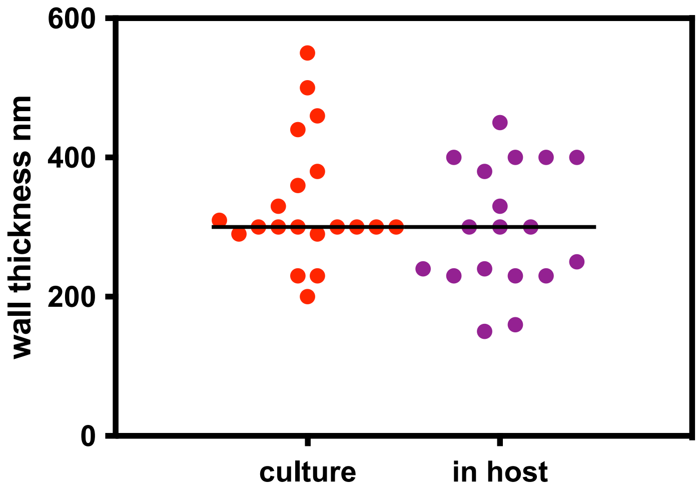

Figure S2. Cell wall thickness is comparable in free-living *B. minutum* cells versus *in hospite* cells.

Scatter plot of wall thickness for 20 free-living cells (culture) and 20 *in hospite* cells measured by transmission electron microscopy. The medians (300nm) are shown by a black line.

Fig. S3 Immunofluorescence detection of *Bm*SWEET1 in dinoflagellates grown at 15 µmol $m^{-2}S^{-1}$ photons (A, B, E & F) versus 60 µmol $m^{-2}S^{-1}$ photons (C, D, G & H) confirming no significant impact on *Bm*SWEET1 abundance due to light intensity in the free living, *in vitro* cultured algae.

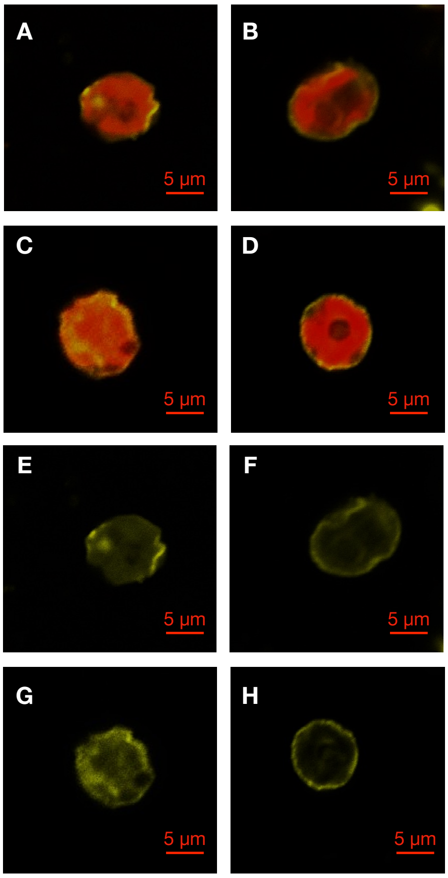

Supplement: Supplementary file 1 — Supplemental material [file 43705_2023_218_MOESM1_ESM.pdf]
